# Therapeutic Effects of Plant Anthocyanin against Alzheimer’s Disease and Modulate Gut Health, Short-Chain Fatty Acids

**DOI:** 10.3390/nu16111554

**Published:** 2024-05-21

**Authors:** Al Borhan Bayazid, Beong Ou Lim

**Affiliations:** 1 Medicinal Biosciences, Department of Applied Biological Sciences, Graduate School, BK21 Program, Konkuk University, Chungju 27478, Republic of Korea; 2 Human Bioscience Corporate R&D Center, Human Bioscience Corp., 268 Chungwondaero, Chungju 27478, Republic of Korea

**Keywords:** anthocyanins, plant bioactive, cognition, neuroprotection, SCFAs, Aβ and tau

## Abstract

Alzheimer’s disease (AD) is the most common form of dementia and neurogenerative disease (NDD), and it is also one of the leading causes of death worldwide. The number of AD patients is over 55 million according to 2020 Alzheimer’s Disease International (ADI), and the number is increasing drastically without any effective cure. In this review, we discuss and analyze the potential role of anthocyanins (ACNs) against AD while understanding the molecular mechanisms. ACNs have been reported as having neuroprotective effects by mitigating cognitive impairments, apoptotic markers, neuroinflammation, aberrant amyloidogenesis, and tauopathy. Taken together, ACNs could be an important therapeutic agent for combating or delaying the onset of AD.

## 1. Introduction

Alzheimer’s disease (AD) is one of the most prevalent neurodegenerative disorders worldwide. It is characterized by progressive cognitive decline, memory loss, and impaired executive functions. With the global population aging rapidly, the burden of AD is expected to escalate significantly, underscoring the urgent need for effective therapeutic strategies [1,2].

**Figure 1:**
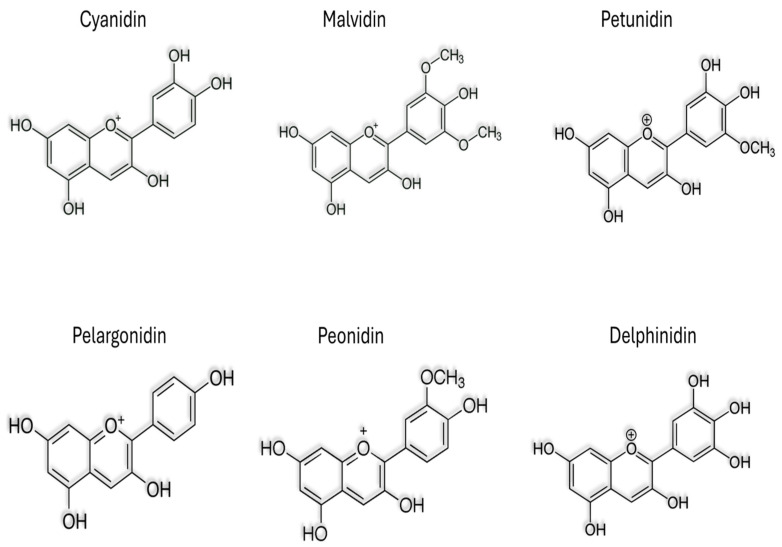
The chemical Structure of Cyanidin, Malvidin, Petunidin, Pelagronidin, Peonidin, and Delphinidin.

In recent years, natural compounds have garnered remarkable attention for their potential effects against neurodegenerative diseases (NDDs), including AD [2,3,4]. Among these, plant-derived anthocyanins (ACNs) have emerged as promising candidates due to their diverse pharmacological properties, including antioxidant, anti-inflammatory, and neuroprotective effects (Graphical Abstract) [4,5,6]. Anthocyanins are water-soluble pigments abundantly found in various fruits, vegetables, flowers, and so on. The major plant ACNs are demonstrated in Figure 1. Beyond their role in plant pigmentation, accumulating evidence suggests that anthocyanins possess remarkable bioactivity with potential health benefits for humans [7,8]. This comprehensive review aims to elucidate the therapeutic effects of plant anthocyanins against AD. We will focus on the ACNs against AD studies and examine their physiological and molecular mechanisms underlying the neuroprotective properties, exploring their ability to modulate key pathways implicated in AD pathology, including oxidative stress, neuroinflammation, amyloid-beta aggregation, and synaptic dysfunction [5,9]. Moreover, this review will provide an overview of preclinical and clinical studies investigating the efficacy of anthocyanin-rich extracts or individual anthocyanin compounds in ameliorating AD-related cognitive impairments and neuropathological alterations. By synthesizing findings in vitro and in vivo, we aim to critically evaluate the therapeutic potential of anthocyanins as a promising adjunctive therapy for AD management. In addition to their direct neuroprotective effects, we will discuss the pharmacokinetics and bioavailability of anthocyanins, considering factors that may influence their efficacy in crossing the blood–brain barrier and exerting therapeutic effects within the central nervous system [10].

Overall, this review seeks to provide a comprehensive understanding of the therapeutic potential of plant anthocyanins in Alzheimer’s disease, offering insights into their mechanisms of action and clinical implications. By elucidating the multifaceted effects of anthocyanins in AD pathogenesis and treatment, we aim to contribute to the development of novel therapeutic strategies for combating this devastating neurological disorder.

## 2. Anthocyanins in Plant

Anthocyanin is a subtype of polyphenol and a major bioactive compound in plants. ACNs are water soluble and also exhibit prominent colors (such as red, purple, violet, etc.) in fruits and vegetables [8,11]. Cyanidin, delphinidin, malvidin, peonidin, petunidin, and pelargonidin are the potent six types of anthocyanidins. Anthocyanins are synthesized in the cytoplasm of plant cells and accumulate in vacuoles, giving bright colors to plant tissues [8,12]. In addition to their aesthetic appeal, anthocyanins also play an important role in plant physiology, including protecting against UV rays, attracting pollinators, and serving as a defense mechanism against germs. diseases and herbivores [12]. Additionally, they are believed to contribute to human health benefits through their antioxidant and anti-inflammatory properties, potentially reducing the risk of chronic diseases [6,13,14,15]. The distribution and concentration of anthocyanins in plants vary widely between species and can be influenced by environmental factors such as light intensity, temperature, and soil conditions. Understanding the biosynthesis and function of anthocyanins in plants not only highlights their ecological importance but also highlights their potential applications in agriculture, nutrition, and medicine. This antioxidant capacity is due to the presence of multiple hydroxyl groups on the anthocyanin molecule, which can donate electrons to neutralize free radicals. Additionally, anthocyanins demonstrate pH-dependent color changes, appearing red in acidic environments and blue or purple in alkaline conditions, a property often utilized in food science and pH indicators [8,13]. ACNs also have UV-light absorbing properties, serving to protect plants from harmful ultraviolet radiation. Furthermore, these pigments may play roles in plant reproduction and defense, attracting pollinators with their vibrant colors and acting as deterrents against herbivores and pathogens. Acylated ACNs can impact the stability, bioavailability, and biological activities of these compounds [14]. Acylation involves the addition of acyl groups (such as acetyl, coumaroyl, or malonyl) to the sugar moiety of anthocyanins, resulting in acylated anthocyanins. Acylation can improve bioavailability and enhance health beneficial effects [14,16]. Previous studies suggest that anthocyanins may offer various health benefits to humans, including cardiovascular protection, anti-inflammatory effects, and potential anticancer properties [6,8,17,18,19]. Overall, the multifaceted properties of anthocyanins make them not only visually appealing but also biologically significant compounds with diverse applications in both nature and human health. The prominent anthocyanins in various fruits and vegetables are mentioned in Table 1 from previous studies.

## 3. Anthocyanins on Gut–Brain Axis

**Figure 2:**
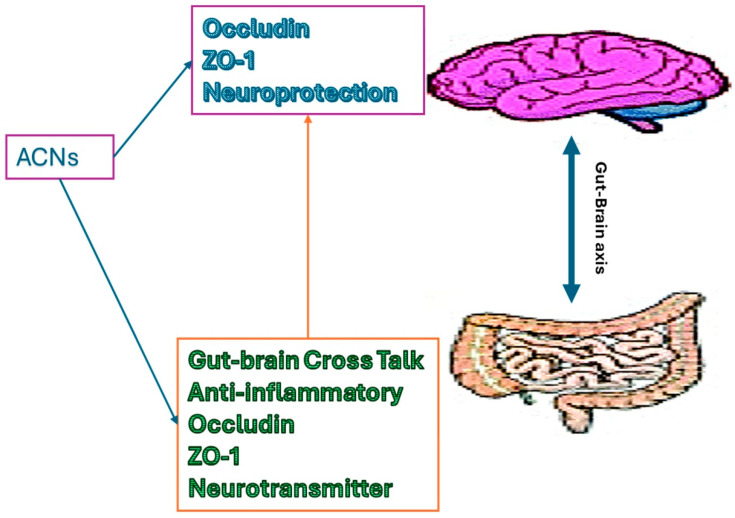
The role of anthocyanins on gut–brain axis.

The gut–brain axis is a bidirectional communication network connecting to the central nervous system (CNS), plays a key role in the pathology of AD [29,30]. Emerging evidence suggests that changes in the composition and function of the gut microbiota may influence neuroinflammation, Aβ deposition, and tau pathology, leading to the progression of AD [30,31,32]. The gut microbiota produces a variety of neurotransmitters and neuroactive compounds that can modulate neurotransmitter signaling in the brain, influencing cognitive function and behavior. Tryptophan, tyrosine, and phenylalanine are the precursors for the serotonin-, dopamine-, and norepinephrine-like potent neurotransmitters, respectively. These are produced in the gut and crosstalk via the vagus nerve to the CNS [33]. The gut also produces short-chain fatty acids (SCFAs), which are immensely studied as being bioactive in neurology [33,34]. Disruptions in the integrity of the intestinal barrier can lead to the translocation of gut-derived toxins and inflammatory molecules into the bloodstream, causing systemic inflammation and potentially worsening the immune system, aggravating neuroinflammation and neuronal damage [31,34,35]. The dysbiosis in the gut triggers inflammatory cytokines and mediators, such as tumor necrosis factor alpha (TNF-α), interferon gamma (IFN-γ), interleukins (ILs), lipopolysaccharides (LPS), making a leaky gut. A leaky gut allows inflammatory cytokines and mediators into the bloodstream and the enteric nervous system, then into the peripheral nervous system (PNS) to the central nervous system (CNS), which may lead to chronic inflammation and disrupt the blood–brain barrier (BBB) [34]. Compromised BBB allows antigens, pathogens, and inflammatory mediators into the CNS and damages the brain cells. Both animal and human studies have indicated that dysbiosis, characterized by an imbalance in the composition and activity of the microbiota, could be detrimental to normal physiological gut health. This disruption may contribute to the development of chronic diseases such as obesity, cardiovascular disease (CVD), and NDDs [17]. Additionally, the gut microbiota is involved in the metabolism of dietary components, including nutrients and polyphenols, which may have a neuroprotective effect against AD. Therefore, targeting the gut–brain axis through dietary, probiotic, or prebiotic interventions is a promising treatment for Alzheimer’s disease due to modulating neuroinflammation, neurotransmitter signaling, and gut microbiota composition to enhance brain health and cognitive function. Recent studies suggest that anthocyanins possess beneficial effects on gut microbiota compositions and functions, promoting the growth of beneficial bacteria while inhibiting the proliferation of harmful species [36,37]. This modulation of the gut microbiota can influence neurotransmitter production and signaling along the gut–brain axis, potentially impacting cognitive function and behavior [36,38]. Additionally, anthocyanins have been shown to inhibit the aggregation of amyloid-beta peptides and tau protein phosphorylation, two hallmark features of AD pathology which are strongly associated with the gut–brain axis [30,38]. Studies have shown that anthocyanins can enhance gut barrier integrity by promoting the expression of tight junction proteins (Occludin, Claudin, ZO-1), which help maintain the integrity of the gut epithelial barrier, thus reducing the translocation of harmful substances from the gut lumen into the bloodstream [39,40] (Figure 2). Additionally, anthocyanins exhibit prebiotic-like effects by selectively promoting the growth of beneficial gut bacteria such as *Bifidobacteria* and *Lactobacilli* while inhibiting the growth of pathogenic bacteria [41]. This modulation of the gut microbiota can contribute to a balanced gut microbial community, which is essential for overall gut health and immune function. Furthermore, the metabolites produced by gut bacteria from anthocyanin fermentation may also exert beneficial effects on gut barrier functions and overall health. Thus, through their actions on both gut barrier functions and gut microbiota composition, ACNs demonstrate potential as therapeutic agents for promoting gastrointestinal health and preventing various gut-related disorders. Thus, through their effects on the gut–brain axis and their neuroprotective properties, anthocyanins represent a promising avenue for the prevention and treatment of AD.

## 4. Anthocyanins on SCFAs

Acetate, propionate, and butyrate are potent SCFAs that play a significant role in maintaining gut health, and they have been implicated in potential therapeutic strategies against AD and other NDDs [42]. SCFAs are primarily produced through the fermentation of undigested carbohydrates (e.g., dietary fibers) by gut microbiota and have been shown to exert anti-inflammatory, antioxidant, and neuroprotective effects. In the context of AD and other NDDs [34,43], dysbiosis of the gut microbiota and increased intestinal permeability are often observed, leading to systemic inflammation and neuroinflammation, which are key contributors to disease progression [29]. SCFAs have been found to regulate gut barrier function, reduce inflammation, and modulate immune responses, thereby potentially mitigating the inflammatory processes implicated in the pathogenesis of AD and other NDDs [44]. Moreover, SCFAs can cross the blood–brain barrier and exert direct effects on brain function, including neuronal signaling and synaptic plasticity [45]. Studies have demonstrated that SCFAs may promote neurogenesis, enhance cognitive function, and protect against neurodegeneration in animal models of AD and other NDDs [32,34,45]. The interplay of anthocyanins, which are abundant in colorful fruits and vegetables, and SCFAs presents a promising avenue for understanding their potential protective mechanisms against AD [39]. ACNs have been shown to influence SCFA production by modulating the gut microbiota composition, favoring the growth of SCFA-producing bacteria [39,46]. SCFAs, such as acetate, propionate, and butyrate, are crucial metabolites that regulate gut barrier function [44,46], reduce systemic inflammation, and potentially exert neuroprotective effects by crossing the blood–brain barrier and modulating brain function [44,47,48]. This interaction may contribute to the observed benefits of anthocyanin-rich diets in promoting gut and brain health and could offer a novel approach for AD prevention and treatment. ACNs naturally traverse the lining of the gastrointestinal tract. Enzymes in the small intestine break them down into phenolic aglycone, primarily in the jejunum. They do not reach the colon intact but are processed by gut bacteria there into simpler forms. These simpler forms have been found to impact the growth of beneficial bacteria like *bifidobacterium*, *Firmicutes*, *Bacteroidetes*, and the production of SCFAs [17,49]. The impact of a diet rich in ACNs on the concentration of SCFAs in the fecal matter of various subjects was examined. Studies focused on the effects of berries, with one study each investigating the effects of black rice and purple sweet potatoes. It is widely accepted that the fermentation of ACNs contributes to the production of SCFAs, primarily acetate, propionate, and butyric acid [17]. These SCFAs play a role in inhibiting the invasion of enteric pathogens like *Salmonella* spp., *E. coli*, and *Listeria* spp. They achieve this by regulating the expression of bacterial virulence genes and reducing the pH value in the colon lumen [50]. However, further research, including clinical studies, is necessary to fully elucidate these mechanisms and evaluate the therapeutic potential of anthocyanins on SCFAs.

## 5. Effects of Anthocyanins against Cognitive and Memory Impairments

Anthocyanins have received significant attention for their potential role in alleviating cognitive and memory decline associated with aging and other forms of NDDs. Studies have shown that anthocyanins exhibit neuroprotective effects through different mechanisms, helping to improve cognitive function and memory by protecting cell death in brain tissue (e.g., cerebral cortex, hippocampus) [4,51]. One of the main ways anthocyanins can exert their beneficial effects is through their powerful antioxidant properties. By eliminating free radicals and reducing oxidative stress in the brain, anthocyanins help protect neurons from damage, thereby preserving cognitive function. Additionally, anthocyanins have been shown to reduce brain inflammation, which is linked to the progression of neurodegenerative diseases such as Alzheimer’s disease [4]. Anthocyanins can improve neural communication and synaptic plasticity, which are necessary for learning and memory [52,53]. Many previous studies have reported that anthocyanin-rich extracts can increase the expression of proteins involved in synaptic transmission and synaptic plasticity, leading to improved cognitive performance in animal models [4,53,54]. Moreover, anthocyanins have been shown to promote neurogenesis, the process of creating new nerve cells in the brain. This may be especially beneficial in combating age-related cognitive decline and neurodegenerative diseases, as it helps maintain the structural and functional integrity of the brain [4,53]. Overall, there is growing evidence that anthocyanins may offer promising therapeutic potential to combat cognitive and memory disorders. However, additional research, including human clinical trials, is needed to fully elucidate the mechanisms of anthocyanin effects on cognitive function and memory, as well as to help determine dosage and efficacy for treatment purposes.

## 6. Anthocyanins in Neuroinflammation

A plethora of studies have focused on the potential role of anthocyanins in modulating neuroinflammation, especially in the context of neurodegenerative diseases such as AD [2,9,36]. Neuroinflammation is a complex process that involves the overactivation of immune cells in the central nervous system in response to injury, infection, or neurodegeneration [55,56]. Chronic neuroinflammation is a prominent feature of AD and is thought to contribute to disease progression and cognitive decline [54,56]. Several studies have investigated the anti-inflammatory properties of anthocyanins and their potential neuroprotective effects in reducing neuroinflammation associated with AD [13,57]. Anthocyanins have been shown to inhibit the activation of microglia and astrocytes, which are the important immune cells in the brain that play a central role in the neuroinflammatory response. By reducing the production of inflammatory cytokines, chemokines, and ROS, ACNs help reduce neuroinflammation and protect against neuronal damage [6,34,58]. ACNs have been shown to regulate signaling pathways involved in inflammation, such as nuclear factor kappa B (NF-κB), mitogen-activated protein kinase (MAPK), nucleotide-binding oligomerization domain, leucine-rich Repeat and Pyrin domain containing 3 (NLRP3), and secret pro-inflammatory cytokines. ACNs illustrate anti-inflammatory effects by suppressing them [55,56,59]. Inflammation also can be triggered by oxidative stress. Anthocyanins can enhance the activity of endogenous antioxidant enzymes and promote the removal of toxic protein aggregates, thereby contributing to their neuroprotective effects in AD. Preclinical studies using animal models of AD have provided evidence supporting the potential therapeutic benefit of anthocyanins in reducing neuroinflammation and improving cognitive deficits. Dietary supplementation with anthocyanin-rich extracts or consumption of anthocyanin-rich foods has been shown to reduce markers of neuroinflammation, improve synaptic function, and improve cognitive performance in dynamic models [6,34]. Although these results are promising, additional research, including human clinical trials, is needed to fully elucidate the therapeutic potential of anthocyanins in targeting neurological inflammation and slowing the progression of AD. In blocking the expression of inflammatory genes and cytokines, ACNs have been shown to inhibit the activation of the NLRP3 inflammasome, a multiprotein complex involved in the processing and release of inflammatory cytokines such as interleukin-1β (IL-1β), IL-18, and TNF-α [34,43]. By targeting multiple aspects of the inflammatory cascade, cytokine production, and inflammasome activation, anthocyanins offer a multifaceted approach to attenuate neuroinflammation in AD. Further investigation into the specific molecular targets and signaling pathways modulated by anthocyanins may provide valuable information for developing new therapeutic strategies against AD. However, growing evidence suggests that anthocyanins may be a promising dietary intervention to reduce neuroinflammation and protect brain health in AD and other neurodegenerative diseases.

## 7. Anthocyanins in Oxidative Stress

Oxidative stress plays a central role in the pathogenesis of AD through interconnected mechanisms [43,60]. Excessive oxidative stress also can trigger ferroptosis, which is a putative factor in AD investigation [56]. The brain is particularly vulnerable to oxidative damage due to its high oxygen consumption, abundant lipid content, and relatively low levels of antioxidant defenses. In AD, the balance between reactive oxygen species (ROS) production and the cellular antioxidant capacity is disrupted, leading to increased oxidative stress and subsequent neurological damage [34,35,60,61]. Excessive ROS may cause the abnormal metabolism of amyloid-beta (Aβ) peptide and lead to AD [62,63]. Accumulation of Aβ aggregates in the brain leads to the generation of ROS through several pathways, including interactions with metal ions such as copper and iron, activation of microglia and astrocytes, as well as disruption of brain function and mitochondrial function [43,60]. The ROS cause oxidative damage to lipids, proteins, and nucleic acids, thereby contributing to neurological dysfunction and cell death. In addition, mitochondrial dysfunction is the pivotal cause of oxidative stress in AD. Impaired mitochondrial function leads to overproduction of ROS and decreased ATP production, exacerbating cellular integrity and affecting neuronal viability. Mitochondrial dysfunction in AD is exacerbated by Aβ accumulation, tau pathology, and impaired mitochondrial biogenesis and dynamics. In addition to Aβ and mitochondrial dysfunction, other factors contribute to oxidative stress in AD, including neuroinflammation, excitotoxicity, and metal homeostasis. Neuroinflammation, characterized by the activation of microglia and astrocytes as well as the release of inflammatory cytokines and chemokines, leads to ROS production and exacerbation of oxidative damage in the brain [34,64]. Excitotoxicity, due to excessive glutamate release and calcium influx, further increases oxidative stress by promoting ROS production and mitochondrial dysfunction [65]. On the other hand, the dysregulation of metal ions, such as copper, iron, and zinc, leads to the generation of ROS through Fenton and Haber–Weiss reactions [66], thereby contributing to oxidative damage in AD. Overall, oxidative stress in AD results from a complex interaction of several factors, including Aβ accumulation, mitochondrial dysfunction, neuroinflammation, excitotoxicity, and ROS imbalance. Targeting oxidative stress is a promising therapeutic strategy for AD, aiming to reduce neuronal damage and slow disease progression. However, the complete mechanisms of oxidative stress in AD remain to be studied in regard to the development of effective antioxidant-based therapies. Anthocyanins have been shown to enhance endogenous antioxidant defenses by upregulating the expression of antioxidant enzymes such as superoxide dismutase (SOD), catalase (CAT), glutathione peroxidase (GPx), and so on [34,67]. By strengthening cellular antioxidant defense systems, anthocyanins help alleviate oxidative stress-induced damage to proteins, lipids, and DNA, which are implicated in the pathogenesis of AD. ACNs exert anti-inflammatory properties, which are closely related to oxidative stress in the context of AD. By blocking inflammatory pathways and reducing the production of inflammatory cytokines and chemokines, anthocyanins help reduce neuroinflammation, a major contributor to oxidative stress and neurodegeneration in AD [2,53,68]. ACNs restore and activate the pivotal Nrf2/HO-1 antioxidant pathways [68,69]. The Nrf2 pathway activation releases the antioxidant enzymes and prevents oxidative stress. Moreover, ACNs possess a unique molecular structure with a conjugated double bond system [18]. This structure allows electrons to be delocalized, enhancing the ability of anthocyanins to scavenge free radicals and ROS. By donating electrons, anthocyanins neutralize these harmful molecules and stabilize resulting radicals, thereby reducing oxidative stress [13,53]. Additionally, the presence of hydroxyl groups in anthocyanins further contributes to their antioxidant activity. Overall, the conjugated double bond system in anthocyanins plays a key role in their protective effects against oxidative damage, highlighting the importance of consuming anthocyanin-rich foods for maintaining health and wellbeing. Preclinical studies have provided evidence supporting the neuroprotective effects of anthocyanins against oxidative stress in animal models of AD. Dietary supplementation with anthocyanin-rich extracts or consumption of anthocyanin-rich foods have been shown to reduce oxidative damage, improve cognitive function, and reduce AD pathology in experimental models [2,13,53]. Overall, the antioxidant properties of anthocyanins make them promising candidates for preventing and treating AD by targeting oxidative stress.

## 8. Protective Effects of Anthocyanins Neuronal Apoptosis

In AD progression, the apoptotic pathway plays a pivotal role in neuronal loss and the pathology of the disease. Aβ aggregates induce oxidative stress, mitochondrial dysfunction, and excitotoxicity, ultimately activating caspase cascades and triggering the intrinsic apoptotic pathway [43,56]. Mitochondrial dysfunction, driven by Aβ accumulation, leads to the release of pro-apoptotic factors such as cytochrome c, which further amplifies caspase activation. Dysregulation of Bcl-2 family proteins, along with neuroinflammation mediated by activated microglia and astrocytes, contributes to apoptotic signaling in neurons [34,61,67]. Additionally, tau pathology, characterized by abnormal phosphorylation and aggregation of tau protein, disrupts microtubule stability and exacerbates apoptotic pathways [43,70]. Accumulating evidence suggests that anthocyanins exert multifaceted protective effects on neurons, mitigating apoptotic pathways implicated in AD pathogenesis [53]. Firstly, anthocyanins possess potent antioxidant properties, scavenging free radicals and reducing oxidative stress, which are known to trigger apoptotic cascades in neurons [71]. Anthocyanins regulate mitochondrial function, an important determinant of programmed cell death. Anthocyanins maintain mitochondrial integrity by stabilizing the mitochondrial membrane potential and preventing the release of apoptotic factors such as cytochrome c. By inhibiting the opening of the mitochondrial permeability transition pore (mPTP) and regulating Bcl-2 family proteins, anthocyanins regulate the intrinsic apoptotic pathway [6,72], which is caused by mitochondrial dysfunction [34,35]. ACNs directly target apoptotic signaling cascades, including caspase activation. These compounds inhibit the activation of caspase-3, a potent factor of apoptosis, and regulate the expression of anti-apoptotic proteins such as Bcl-2 and Bcl-xL, thereby promoting the survival of nerve cells [6,73]. Anthocyanins also regulate apoptosis regulators such as p53, a tumor suppressor protein that coordinates apoptotic responses to cellular stress [53,54]. In general, the protective effects of anthocyanins on the AD apoptotic pathway involve a complex interaction of antioxidant, anti-inflammatory, mitochondrial protective, and direct anti-apoptotic mechanisms. By targeting multiple aspects of the apoptotic cascade, anthocyanins offer promising therapeutic potential to reduce neuronal apoptosis and preserve cognitive function during AD.

## 9. Anthocyanins on Insulin Resistance

Insulin resistance, a hallmark of type 2 diabetes mellitus, has emerged as a significant contributor to AD pathogenesis, referred to as type 3 diabetes [55,74], highlighting the intricate link between metabolic dysfunction and neurodegeneration. In AD, insulin resistance disrupts insulin signaling pathways in the brain, impairing neuronal glucose uptake and metabolism and compromising synaptic function and neuronal survival [55]. Dysregulated insulin signaling leads to neuronal insulin resistance, characterized by reduced responsiveness to insulin stimulation, dysregulated insulin receptor substrate (IRS) phosphorylation, and impaired activation of downstream-signaling cascades such as phosphatidylinositol 3-kinase (PI3K) and Akt/protein kinase B (PKB) pathways [55,64]. Moreover, insulin resistance promotes neuroinflammation, oxidative stress, tauopathy, and Aβ accumulation, and it aggravates the onset of AD pathology [55]. Conversely, AD-associated pathological hallmarks, including Aβ accumulation, tau hyperphosphorylation, and neuroinflammation, further impair insulin signaling, creating a vicious cycle of neurodegeneration and metabolic dysfunction [64,75]. Collectively, insulin resistance in AD underscores the intricate interplay between metabolic dysregulation and neurodegeneration, highlighting the potential therapeutic relevance of targeting insulin signaling pathways for AD prevention and treatment. Previous studies suggest that anthocyanins may exert beneficial effects on insulin signaling pathways, enhancing insulin sensitivity and glucose metabolism in the brain [2,53]. By scavenging free radicals and reducing oxidative stress, anthocyanins protect insulin signaling molecules from damage and preserve their functionality. Furthermore, anthocyanins have been found to modulate inflammatory pathways and inhibit neuroinflammation, a driver of insulin resistance and AD pathogenesis. Anthocyanin-rich diets have been associated with a reduced risk of developing insulin resistance and AD in epidemiological studies [14,40,76,77]. Overall, the neuroprotective effects of anthocyanins on insulin resistance in AD highlight their potential as therapeutic agents for targeting metabolic dysfunction and cognitive decline in the progression of NDDs.

## 10. Effects of Anthocyanins on Neurogenesis

In Alzheimer’s disease and other NDDs, understanding the complex mechanisms underlying neurodegeneration is critical for developing effective treatments. Neurogenesis, the process of creating new nerve cells, plays a central role in brain function and repair [34,35]. However, in AD, neurogenesis is impaired, contributing to cognitive decline and neuronal loss. Brain-derived neurotrophic factor (BDNF) and cAMP response element-binding protein (CREB) are important regulators of neurogenesis and synaptic plasticity, and both are found less in an AD-affected brain [34,78,79]. Peroxisome proliferator-activated receptor gamma (PPAR-γ) has emerged as a promising therapeutic target in AD due to its role in neuroprotection, anti-inflammatory elements, and the promotion of neurogenesis [69,80]. Understanding the complex interactions between these factors provides insight into the pathophysiology of AD and may pave the way for novel therapeutic interventions aimed at promoting neurogenesis and preserving cognitive function. Recent research suggests that anthocyanins may play a significant role in promoting neurogenesis, the process of generating new neurons, thereby counteracting the neuronal loss characteristic of AD [3,54]. Moreover, anthocyanins have been shown to upregulate BDNF and CREB, crucial molecules involved in neuronal survival, synaptic plasticity, and memory formation [78,79]. Furthermore, ACNs activate PPAR-γ with anti-inflammatory and neuroprotective properties [69]. By modulating these key pathways, anthocyanins have been demonstrated as potential therapeutic agents for mitigating cognitive decline and neuronal damage in Alzheimer’s disease. Further elucidation of the mechanisms underlying anthocyanin-mediated neuroprotection may pave the way for the development of novel interventions aimed at preserving cognitive function and slowing the progression of AD.

## 11. Role of Anthocyanins in Amyloid-Beta and Tauopathy

Aβ and hyperphosphorylation of tau are the two hallmarks of AD. The staging of AD neuropathology revolves around pinpointing where the pathological changes occur in the brain, evaluated through a semi-quantitative assessment of their density. Present models utilize an ABC scoring system, with stages attributed to Aβ plaques (A), the Braak stage, indicating tau neurofibrillary tangles (B), and the Consortium to Establish a Registry for Alzheimer’s Disease (CERAD) score-assessing neuritic plaques (C) [43,63]. Amyloid-β plaque distribution is categorized into stages according to Thal staging, which progresses through five phases from widespread neocortical deposition to involvement of deeper brain structures like the cerebellum. Tau neurofibrillary tangles are classified using Braak and Braak’s staging system, starting from early stages in specific cortical areas to more widespread involvement across the brain. Neuritic plaques are assessed using the CERAD system, which ranks their density in the neocortex. A novel biomarker classification system for AD proposes three primary biomarker classes: amyloid-β (A), tau (T), and neurodegeneration (N), denoted as A/T/(N) [62,81], which are interrelated. While abnormal Aβ and phosphorylated tau levels are core features of AD [62,70], neurodegeneration is common in other NDDs as well. Aβ can be generated internally within cells or acquired from outside through receptor binding, leading to its accumulation within various cellular compartments [81]. Intracellular Aβ accumulation in transgenic mouse models is associated with cognitive impairment, tau phosphorylation, neuronal loss, and synaptic dysfunction. Despite the prevailing belief that Aβ is primarily deposited outside cells, emerging evidence from both animal models and human patients suggests its intraneuronal accumulation, potentially contributing to disease advancement [81]. Recent studies have revealed that anthocyanins have anti-amyloidogenic and anti-tau properties, suggesting their therapeutic potential in alleviating the pathology characteristics of AD. These compounds demonstrate an ability to inhibit the aggregation of Aβ peptides into toxic oligomers and fibrils, thereby reducing plaque formation in the brain [4,51,54,73]. Anthocyanins have been shown to reduce tau hyperphosphorylation [2,5], a key step in the development of neurofibrillary disorders, by regulating key kinases involved in tau phosphorylation. ACNs, or ACN-rich plant extracts, are also reported as inhibiting Aβ accumulation and neurodegeneration [2,4,5]. Therefore, the consumption of anthocyanin-rich foods or the use of anthocyanin supplements holds promise as a preventive or therapeutic strategy against the progression of AD, providing a natural and accessible approach to combating this debilitating neurodegenerative disease.

## 12. Anthocyanins on Proteostasis

**Figure 3:**
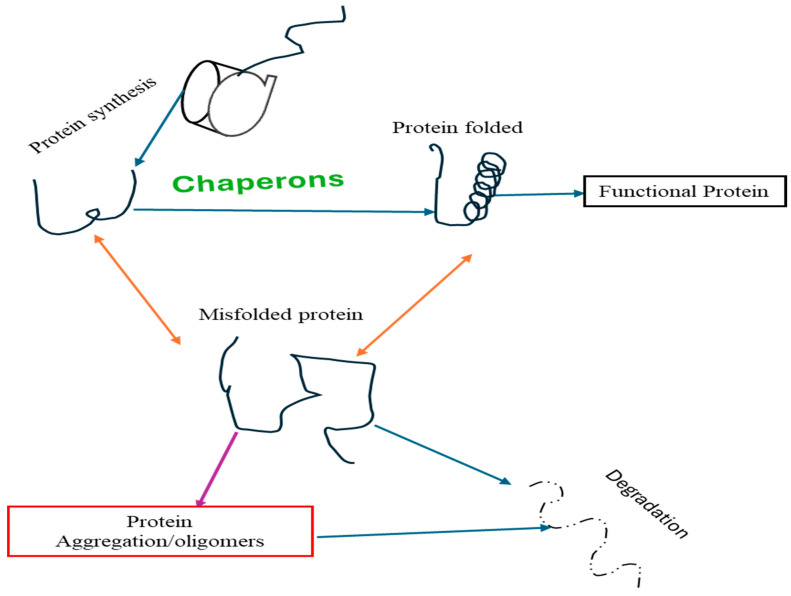
Diagram of Proteostasis.

Proteostasis is the complex balance of protein synthesis, folding, transport, and degradation that plays a critical role in maintaining cellular function and viability [82]. In AD, disruption of proteostasis (Figure 3) leads to the accumulation of misfolded proteins, including Aβ plaques and tau tangles, which contribute to neurological dysfunction and cognitive decline [82,83]. Protein imbalance in AD involves the impairment of various cellular pathways, such as the ubiquitin–proteasome system (UPS), the autophagy–lysosomal pathway (ALP), and chaperone-mediated protein folding [71,84]. Dysfunction of these pathways results in an inability to appropriately remove misfolded proteins, leading to their aggregation and toxicity. The changes in molecular chaperones, heat shock proteins, and proteases further exacerbate protein dysregulation in AD [82]. Understanding the complex interplay of proteomic mechanisms in AD pathogenesis is critical for developing targeted therapeutic strategies to restore proteostasis and attenuate aggregation-induced neurotoxicity caused by proteins [83]. Therapeutic interventions aimed at protein stabilization show promise in slowing disease progression and preserving cognitive function in people with AD. Studies have shown that anthocyanins contribute to protein misfolding and synthesis [85,86]. ACNs have been found to enhance the activity of proteolytic systems involved in protein clearance, such as the UPS and ALP [87]. By promoting the clearance of misfolded proteins, including Aβ and tau, anthocyanins may help reduce protein dysregulation and reduce neuronal damage in AD. ACNs have been shown to regulate molecular chaperones and heat shock proteins, which play important roles in protein folding and degradation. Overall, the multifaceted effects of anthocyanins on proteostasis highlight their potential as therapeutic agents targeting protein aggregation and neurodegeneration in AD. Further investigation into the specific mechanisms of anthocyanin-mediated modulation of protein stability is needed to fully elucidate their therapeutic potential in regard to AD.

## 13. Effects of Anthocyanins on Epigenetics of Alzheimer’s Disease

The epigenetics of AD involves complex changes in chromatin structure and gene regulation that contribute to disease pathogenesis. Histone modifications, such as acetylation, methylation, phosphorylation, and ubiquitination [88,89,90], play an important role in regulating chromatin accessibility and the transcriptional activity of genes involved in AD. Dysregulation of histone acetylation, specifically decreased histone acetyltransferase (HAT) activity and increased histone deacetylase (HDAC) activity, has been observed in AD-affected brains [88]. This leads to aberrant gene expression patterns associated with neurodegeneration in the cerebral cortex and hippocampus, which leads to cognitive decline [55,91]. Histone methylation, including lysine and arginine methylation, also affects AD-related gene expression through the recruitment of chromatin-modifying enzymes and transcriptional regulators [92]. DNA methylation is a well-studied epigenetic modification that is dynamically regulated in the AD brain and contributes to the inactivation of genes involved in synaptic plasticity and inflammation. Additionally, non-coding RNAs, such as microRNAs and long non-coding RNAs, participate in epigenetic regulation by regulating mRNA stability and translation of AD-related genes [89]. Understanding the complex interplay of epigenetic mechanisms, including histone modifications and DNA methylation, in the pathogenesis of AD is critical to elucidating pathogenic mechanisms and identifying targets. Further research into the epigenetic landscape of AD promises to develop new diagnostic biomarkers and epigenetic therapies aimed at stopping or reversing disease progression. Research on the epigenetic effects of anthocyanins in AD is still developing, but there is evidence suggesting their potential to regulate histone modifications, DNA methylation, and other epigenetic mechanisms involved in disease pathogenesis [93]. Anthocyanins can influence epigenetic processes. Histone modifications: many anthocyanins have been shown to influence histone modification patterns. For example, studies have demonstrated that certain anthocyanins can inhibit HDAC activity, leading to increased levels of histone acetylation [94]. This change in histone acetylation status may influence gene expression patterns related to neuroprotection and synaptic plasticity, which may influence AD pathology [9]. Anthocyanin can also regulate DNA methylation, another important epigenetic mechanism. Some studies show that anthocyanins can alter DNA methyltransferase (DNMT) activity, leading to changes in DNA methylation patterns. By influencing DNA methylation, anthocyanins may regulate the expression of genes involved in AD pathogenesis, such as genes involved in amyloid processing, inflammation, and neuronal function [2,95]. By regulating the expression or activity of these non-coding RNAs, anthocyanins may indirectly influence the expression of AD-related epigenetic pathways. Although there is growing evidence supporting the epigenetic effects of anthocyanins, additional research is needed to fully elucidate the mechanisms by which they influence epigenetic processes in the context of AD.

## 14. Conclusions

Anthocyanins are the major bioactive element in color fruits and vegetables that involve safe usage. ACNs illustrate a plethora of health-beneficial effects by their enormous antioxidant, anti-inflammatory, anti-aging, anti-cancer, anti-obesity, and many other bio-functional effects. Recently, ACNs have gained heightened attention for their neurological and psychiatric effects. ACNs improve cognitive impairment, neuronal cell loss, neuroinflammation, and so on. They also facilitate proteostasis, gut health, epigenetic factors, and neurogenesis to protect against the progression of AD. However, ACNs have been reported to have low bioavailability, and there is no clinical study reported regarding ACNs being used against AD. Concurrently, ACNs significantly reduce amyloid-beta, and tau hyperphosphorylation, which are the hallmarks of AD. Moreover, ACNs reverse insulin resistance, modulate gut–brain axis and cognition, etc. Taken together, ACNs could be a promising therapeutic candidate for clinical trials against AD or other forms of dementia.

## Figures and Tables

**Table 1:** Anthocyanins in various vegetables and fruits.

Source	Content	Refs.
Red Grape	Cyanidin-3-glucoside, Delphinidin-3-glucoside, Malvidin-3-glucoside, Peonidin-3-glucoside	[20]
Blackcurrant	Delphinidin-3-rutinoside, Delphinidin-3-glucoside, Cyanidin-3-rutinoside, Cyanidin-3-glucoside	[21,22]
Purple Potato	Petunidin glucoside, Peonidin glucoside, Malvidin glucoside	[14]
Eggplant	Delphinidin 3-O-rutinoside-5-glucoside, Delphinidin 3-O-glucoside, Cyanidin 3-O-rutinoside	[23]
Red Cabbage	Cyanidin-3-diglucoside-5-glucoside.	[24]
Blueberry	Delphinidin 3-galactoside, Cyanidin 3-galactoside, Cyanidin 3-arabinoside, Peonidin 3-galactoside, Peonidin 3-arabinoside	[25]
Blackberry	Naringenin-7-O-glucoside, Quercetin-3-O-glucoside, Kaempferol-3-O-rutinoside	[26]
Raspberry	Cyanidins, Pelargonidins	[27]
Strawberry	Peonidin-3- glucoside, Peonidin-3-rutinoside, Cyanidin-3-glucoside	[7]
Cherry	Cyanidin-3-O-glucoside, Cyanidin-3-O-rutinoside	[28]

## References

[1] Krikorian R., Nash T.A., Shidler M.D., Shukitt-Hale B., Joseph J.A. (2010). Concord grape juice supplementation improves memory function in older adults with mild cognitive impairment. Br. J. Nutr..

[2] Suresh S., Begum R.F., Singh S A., V C. (2022). Anthocyanin as a therapeutic in Alzheimer’s disease: A systematic review of preclinical evidences. Ageing Res. Rev..

[3] Gao J., Wu Y., He D., Zhu X., Li H., Liu H., Liu H. (2020). Anti-aging effects of Ribes meyeri anthocyanins on neural stem cells and aging mice. Aging.

[4] Subash S., Essa M.M., Al-Adawi S., Memon M.A., Manivasagam T., Akbar M. (2014). Neuroprotective effects of berry fruits on neurodegenerative diseases. Neural Regen. Res..

[5] Ali T., Kim M.J., Rehman S.U., Ahmad A., Kim M.O. (2017). Anthocyanin-Loaded PEG-Gold Nanoparticles Enhanced the Neuroprotection of Anthocyanins in an Aβ1–42 Mouse Model of Alzheimer’s Disease. Mol. Neurobiol..

[6] Winter A.N., Bickford P.C. (2019). Anthocyanins and Their Metabolites as Therapeutic Agents for Neurodegenerative Disease. Antioxidants.

[7] Da Silva F.L., Escribano-Bailón M.T., Pérez Alonso J.J., Rivas-Gonzalo J.C., Santos-Buelga C. (2007). Anthocyanin pigments in strawberry. LWT-Food Sci. Technol..

[8] Welch C.R., Wu Q., Simon J.E. (2008). Recent Advances in Anthocyanin Analysis and Characterization. Curr. Anal. Chem..

[9] Afzal M., Redha A., AlHasan R. (2019). Anthocyanins Potentially Contribute to Defense against Alzheimer’s Disease. Molecules.

[10] Shimazu R., Anada M., Miyaguchi A., Nomi Y., Matsumoto H. (2021). Evaluation of Blood–Brain Barrier Permeability of Polyphenols, Anthocyanins, and Their Metabolites. J. Agric. Food Chem..

[11] Menconi J., Perata P., Gonzali S. (2024). In pursuit of purple: Anthocyanin biosynthesis in fruits of the tomato clade. Trends Plant Sci..

[12] Jiang Y., Li X., Zhang Y., Wu B., Li Y., Tian L., Sun J., Bai W. (2024). Mechanism of action of anthocyanin on the detoxification of foodborne contaminants—A review of recent literature. Compr. Rev. Food Sci. Food Saf..

[13] Mohammadi N., Farrell M., O’Sullivan L., Langan A., Franchin M., Azevedo L., Granato D. (2024). Effectiveness of anthocyanin-containing foods and nutraceuticals in mitigating oxidative stress, inflammation, and cardiovascular health-related biomarkers: A systematic review of animal and human interventions. Food Funct..

[14] Jokioja J., Linderborg K.M., Kortesniemi M., Nuora A., Heinonen J., Sainio T., Viitanen M., Kallio H., Yang B. (2020). Anthocyanin-rich extract from purple potatoes decreases postprandial glycemic response and affects inflammation markers in healthy men. Food Chem..

[15] Bayazid A.B., Chun E.M., Mijan M.A., Park S.H., Moon S.-K., Lim B.O. (2021). Anthocyanins Profiling of Bilberry (*Vaccinium myrtillus* L.) Extract that Elucidates Antioxidant and Anti-inflammatory Effects. Food Agric. Immunol..

[16] Jokioja J., Yang B., Linderborg K.M. (2021). Acylated anthocyanins: A review on their bioavailability and effects on postprandial carbohydrate metabolism and inflammation. Compr. Rev. Food Sci. Food Saf..

[17] Liang A., Leonard W., Beasley J.T., Fang Z., Zhang P., Ranadheera C.S. (2023). Anthocyanins-gut microbiota-health axis: A review. Crit. Rev. Food Sci. Nutr..

[18] Khoo H.E., Azlan A., Tang S.T., Lim S.M. (2017). Anthocyanidins and anthocyanins: Colored pigments as food, pharmaceutical ingredients, and the potential health benefits. Food Nutr. Res..

[19] Rahhal B., Qneibi M., Jaradat N., Hawash M., Qadi M., Issa L., Bdir S. (2024). Multi-biological activity assessment and phytochemical characterization of an aqueous extract of the Cymbopogon citratus grown in Palestine. BMC Complement. Med. Ther..

[20] Bitsch R., Netzel M., Frank T., Strass G., Bitsch I. (2004). Bioavailability and Biokinetics of Anthocyanins From Red Grape Juice and Red Wine. J. Biomed. Biotechnol..

[21] Braakhuis A.J., Somerville V.X., Hurst R.D. (2020). The effect of New Zealand blackcurrant on sport performance and related biomarkers: A systematic review and meta-analysis. J. Int. Soc. Sports Nutr..

[22] Hollands W., Brett G.M., Radreau P., Saha S., Teucher B., Bennett R.N., Kroon P.A. (2008). Processing blackcurrants dramatically reduces the content and does not enhance the urinary yield of anthocyanins in human subjects. Food Chem..

[23] Condurache N.-N., Croitoru C., Enachi E., Bahrim G.-E., Stănciuc N., Râpeanu G. (2021). Eggplant Peels as a Valuable Source of Anthocyanins: Extraction, Thermal Stability and Biological Activities. Plants.

[24] Ghareaghajlou N., Hallaj-Nezhadi S., Ghasempour Z. (2021). Red cabbage anthocyanins: Stability, extraction, biological activities and applications in food systems. Food Chem..

[25] Yang W., Guo Y., Liu M., Chen X., Xiao X., Wang S., Gong P., Ma Y., Chen F. (2022). Structure and function of blueberry anthocyanins: A review of recent advances. J. Funct. Foods.

[26] Li J., Shi C., Shen D., Han T., Wu W., Lyu L., Li W. (2022). Composition and Antioxidant Activity of Anthocyanins and Non-Anthocyanin Flavonoids in Blackberry from Different Growth Stages. Foods.

[27] Teng H., Fang T., Lin Q., Song H., Liu B., Chen L. (2017). Red raspberry and its anthocyanins: Bioactivity beyond antioxidant capacity. Trends Food Sci. Technol..

[28] Grigoras C.G., Destandau E., Zubrzycki S., Elfakir C. (2012). Sweet cherries anthocyanins: An environmental friendly extraction and purification method. Sep. Purif. Technol..

[29] Yang C., Xue J., Qin Q., Xia Y., Cheng S., Jiang X., Zhang S., Lu Z., Qin X., Zhang J. (2022). Prenatal exposure to titanium dioxide nanoparticles induces persistent neurobehavioral impairments in maternal mice that is associated with microbiota-gut-brain axis. Food Chem. Toxicol..

[30] Aburto M.R., Cryan J.F. (2024). Gastrointestinal and brain barriers: Unlocking gates of communication across the microbiota–gut–brain axis. Nat. Rev. Gastroenterol. Hepatol..

[31] Doifode T., Giridharan V.V., Generoso J.S., Bhatti G., Collodel A., Schulz P.E., Forlenza O.V., Barichello T. (2021). The impact of the microbiota-gut-brain axis on Alzheimer’s disease pathophysiology. Pharmacol. Res..

[32] Xie J., Bruggeman A., De Nolf C., Vandendriessche C., Van Imschoot G., Van Wonterghem E., Vereecke L., Vandenbroucke R.E. (2023). Gut microbiota regulates blood-cerebrospinal fluid barrier function and Aβ pathology. EMBO J..

[33] Chen Y., Xu J., Chen Y. (2021). Regulation of Neurotransmitters by the Gut Microbiota and Effects on Cognition in Neurological Disorders. Nutrients.

[34] Bayazid A.B., Kim J.G., Azam S., Jeong S.A., Kim D.H., Park C.W., Lim B.O. (2022). Sodium butyrate ameliorates neurotoxicity and exerts anti-inflammatory effects in high fat diet-fed mice. Food Chem. Toxicol..

[35] Bayazid A.B., Jeong S.A., Azam S., Oh S.H., Lim B.O. (2023). Neuroprotective Effects of Fermented Blueberry and Black Rice against Particulate Matter 2.5 μm-Induced Inflammation In Vitro and In Vivo. Preprints.

[36] Zhang N., Jing P. (2023). Red Cabbage Anthocyanins Attenuate Cognitive Impairment By Attenuating Neuroinflammation and Regulating Gut Microbiota in Aging Mice. J. Agric. Food Chem..

[37] Igwe E.O., Roodenrys S., Probst Y.C., do Rosario V., Netzel M.E., Hong H.T., Netzel G., Phan A.D.T., Charlton K.E. (2020). Low anthocyanin plum nectar does not impact cognition, blood pressure and gut microbiota in healthy older adults: A randomized crossover trial. Nutr. Res..

[38] Khan M.S., Ikram M., Park J.S., Park T.J., Kim M.O. (2020). Gut Microbiota, Its Role in Induction of Alzheimer’s Disease Pathology, and Possible Therapeutic Interventions: Special Focus on Anthocyanins. Cells.

[39] Jamar G., Estadella D., Pisani L.P. (2017). Contribution of anthocyanin-rich foods in obesity control through gut microbiota interactions. BioFactors.

[40] Cremonini E., Daveri E., Mastaloudis A., Adamo A.M., Mills D., Kalanetra K., Hester S.N., Wood S.M., Fraga C.G., Oteiza P.I. (2019). Anthocyanins protect the gastrointestinal tract from high fat diet-induced alterations in redox signaling, barrier integrity and dysbiosis. Redox Biol..

[41] Jayarathne S., Stull A.J., Park O.-H., Kim J.H., Thompson L., Moustaid-Moussa N. (2019). Protective Effects of Anthocyanins in Obesity-Associated Inflammation and Changes in Gut Microbiome. Mol. Nutr. Food Res..

[42] Dong Y., Cui C. (2022). The role of short-chain fatty acids in central nervous system diseases. Mol. Cell. Biochem..

[43] Bayazid A.B., Jeong Y.H., Jeong S.A., Lim B.O. (2023). Sodium butyrate alleviates potential Alzheimer’s disease in vitro by suppressing Aβ and tau activation and ameliorates Aβ-induced toxicity. Food Agric. Immunol..

[44] Parada Venegas D., De la Fuente M.K., Landskron G., González M.J., Quera R., Dijkstra G., Harmsen H.J.M., Faber K.N., Hermoso M.A. (2019). Short Chain Fatty Acids (SCFAs)-Mediated Gut Epithelial and Immune Regulation and Its Relevance for Inflammatory Bowel Diseases. Front. Immunol..

[45] He X., Zhang T., Zeng Y., Pei P., Liu Y., Jia W., Zhao H., Bi M., Wang S. (2022). Sodium butyrate mediates histone crotonylation and alleviated neonatal rats hypoxic–ischemic brain injury through gut–brain axis. Front. Microbiol..

[46] Tian B., Zhao J., Zhang M., Chen Z., Ma Q., Liu H., Nie C., Zhang Z., An W., Li J. (2021). Lycium ruthenicum Anthocyanins Attenuate High-Fat Diet-Induced Colonic Barrier Dysfunction and Inflammation in Mice by Modulating the Gut Microbiota. Mol. Nutr. Food Res..

[47] Zhao R., Shen G.X. (2023). Impact of anthocyanin component and metabolite of Saskatoon berry on gut microbiome and relationship with fecal short chain fatty acids in diet-induced insulin resistant mice. J. Nutr. Biochem..

[48] Hu S., Lin Z., Zhao S., Zhang B., Luo L., Zeng L. (2023). Pu-erh tea alleviated colitis-mediated brain dysfunction by promoting butyric acid production. Food Chem. Toxicol..

[49] Kapoor P., Tiwari A., Sharma S., Tiwari V., Sheoran B., Ali U., Garg M. (2023). Effect of anthocyanins on gut health markers, Firmicutes-Bacteroidetes ratio and short-chain fatty acids: A systematic review via meta-analysis. Sci. Rep..

[50] Sun Y., O’Riordan M.X.D., Sariaslani S., Gadd G.M. (2013). Chapter Three—Regulation of Bacterial Pathogenesis by Intestinal Short-Chain Fatty Acids. Advances in Applied Microbiology.

[51] do Rosario V.A., Fitzgerald Z., Broyd S., Paterson A., Roodenrys S., Thomas S., Bliokas V., Potter J., Walton K., Weston–Green K. (2021). Food anthocyanins decrease concentrations of TNF-α in older adults with mild cognitive impairment: A randomized, controlled, double blind clinical trial. Nutr. Metab. Cardiovasc. Dis..

[52] Carbonel A.A.F., Cecyn M.N., Girão J.H.R.C., da Silva Sasso G.R., de Mello Ponteciano B., Vellozo E.P., Simões R.S., de Jesus Simões M., Girão M.J.B.C., de Oliveira D.R. (2019). Flavonoids as Modulators of Synaptic Plasticity: Implications for the Development of Novel Therapeutic Strategies for Healthy Lifestyle. Flavonoids—A Coloring Model for Cheering up Life.

[53] Rehman S.U., Shah S.A., Ali T., Chung J.I., Kim M.O. (2017). Anthocyanins Reversed D-Galactose-Induced Oxidative Stress and Neuroinflammation Mediated Cognitive Impairment in Adult Rats. Mol. Neurobiol..

[54] Milenkovic D., Krga I., Dinel A.-L., Morand C., Laye S., Castanon N. (2021). Nutrigenomic modification induced by anthocyanin-rich bilberry extract in the hippocampus of ApoE-/- mice. J. Funct. Foods.

[55] Xu J., Gao H., Zhang L., Rong S., Yang W., Ma C., Chen M., Huang Q., Deng Q., Huang F. (2019). Melatonin alleviates cognition impairment by antagonizing brain insulin resistance in aged rats fed a high-fat diet. J. Pineal Res..

[56] Mangalmurti A., Lukens J.R. (2022). How neurons die in Alzheimer’s disease: Implications for neuroinflammation. Curr. Opin. Neurobiol..

[57] Dong G., Xu N., Wang M., Zhao Y., Jiang F., Bu H., Liu J., Yuan B., Li R. (2021). Anthocyanin Extract from Purple Sweet Potato Exacerbate Mitophagy to Ameliorate Pyroptosis in Klebsiella pneumoniae Infection. Int. J. Mol. Sci..

[58] Davinelli S., Maes M., Corbi G., Zarrelli A., Willcox D.C., Scapagnini G. (2016). Dietary phytochemicals and neuro-inflammaging: From mechanistic insights to translational challenges. Immun. Ageing.

[59] Bayazid A.B., Hwang U.K., Jang Y.A., Jeong Y.H., Jo Y.C., Lim B.O. (2024). Andrographis paniculata Leaves Extract Alleviates UVB-Induced HaCaT Cells Through Suppressing Mitogen-Activated Protein Kinases Activation. Nat. Prod. Commun..

[60] Butterfield D.A., Halliwell B. (2019). Oxidative stress, dysfunctional glucose metabolism and Alzheimer disease. Nat. Rev. Neurosci..

[61] Cox D., Ormsby A.R., Reid G.E., Hatters D.M. (2022). Protein painting reveals pervasive remodeling of conserved proteostasis machinery in response to pharmacological stimuli. NPJ Syst. Biol. Appl..

[62] Jack C.R., Bennett D.A., Blennow K., Carrillo M.C., Feldman H.H., Frisoni G.B., Hampel H., Jagust W.J., Johnson K.A., Knopman D.S. (2016). A/T/N: An unbiased descriptive classification scheme for Alzheimer disease biomarkers. Neurology.

[63] Therriault J., Zimmer E.R., Benedet A.L., Pascoal T.A., Gauthier S., Rosa-Neto P. (2022). Staging of Alzheimer’s disease: Past, present, and future perspectives. Trends Mol. Med..

[64] Wei Z., Koya J., Reznik S.E. (2021). Insulin Resistance Exacerbates Alzheimer Disease via Multiple Mechanisms. Front. Neurosci..

[65] Weilinger N.L., Lohman A.W., Rakai B.D., Ma E.M.M., Bialecki J., Maslieieva V., Rilea T., Bandet M.V., Ikuta N.T., Scott L. (2016). Metabotropic NMDA receptor signaling couples Src family kinases to pannexin-1 during excitotoxicity. Nat. Neurosci..

[66] Malosio M.L., Tecchio F., Squitti R. (2021). Molecular mechanisms underlying copper function and toxicity in neurons and their possible therapeutic exploitation for Alzheimer’s disease. Aging Clin. Exp. Res..

[67] Cho E., Lee J., Sin J.S., Kim S.-k., Kim C.J., Park M.H., Cho W.-S., Moon M., Kim D.H., Jung J.W. (2022). Effects of Perilla frutescens var. acuta in amyloid β toxicity and Alzheimer’s disease-like pathology in 5XFAD mice. Food Chem. Toxicol..

[68] Luo J.-F., Shen X.-Y., Lio C.K., Dai Y., Cheng C.-S., Liu J.-X., Yao Y.-D., Yu Y., Xie Y., Luo P. (2018). Activation of Nrf2/HO-1 Pathway by Nardochinoid C Inhibits Inflammation and Oxidative Stress in Lipopolysaccharide-Stimulated Macrophages. Front. Pharmacol..

[69] Aboonabi A., Aboonabi A. (2020). Anthocyanins reduce inflammation and improve glucose and lipid metabolism associated with inhibiting nuclear factor-kappaB activation and increasing PPAR-γ gene expression in metabolic syndrome subjects. Free. Radic. Biol. Med..

[70] Mancuso R., Fattorelli N., Martinez-Muriana A., Davis E., Wolfs L., Van Den Daele J., Geric I., Premereur J., Polanco P., Bijnens B. (2024). Xenografted human microglia display diverse transcriptomic states in response to Alzheimer’s disease-related amyloid-β pathology. Nat. Neurosci..

[71] Jaber M., Shawahna R., Abu-Issa M., Radwan F., Dweik M. (2021). Anesthesia considerations for patients with epilepsy: Findings of a qualitative study in the Palestinian practice. Epilepsy Behav..

[72] Bernardi P., Gerle C., Halestrap A.P., Jonas E.A., Karch J., Mnatsakanyan N., Pavlov E., Sheu S.-S., Soukas A.A. (2023). Identity, structure, and function of the mitochondrial permeability transition pore: Controversies, consensus, recent advances, and future directions. Cell Death Differ..

[73] Shih P.-H., Wu C.-H., Yeh C.-T., Yen G.-C. (2011). Protective Effects of Anthocyanins against Amyloid β-Peptide-Induced Damage in Neuro-2A Cells. J. Agric. Food Chem..

[74] Kshirsagar V., Thingore C., Juvekar A. (2021). Insulin resistance: A connecting link between Alzheimer’s disease and metabolic disorder. Metab. Brain Dis..

[75] Kellar D., Craft S. (2020). Brain insulin resistance in Alzheimer’s disease and related disorders: Mechanisms and therapeutic approaches. Lancet Neurol..

[76] Ye X., Chen W., Huang X.-F., Yan F.-J., Deng S.-G., Zheng X.-D., Shan P.-F. (2024). Anti-diabetic effect of anthocyanin cyanidin-3-O-glucoside: Data from insulin resistant hepatocyte and diabetic mouse. Nutr. Diabetes.

[77] de Mello J.E., Teixeira F.C., dos Santos A., Luduvico K., Soares de Aguiar M.S., Domingues W.B., Campos V.F., Tavares R.G., Schneider A., Stefanello F.M. (2024). Treatment with Blackberry Extract and Metformin in Sporadic Alzheimer’s Disease Model: Impact on Memory, Inflammation, Redox Status, Phosphorylated Tau Protein and Insulin Signaling. Mol. Neurobiol..

[78] Ridzwan N., Jumli M.N., Baig A.A., Rohin M.A.K. (2020). Pomegranate-derived anthocyanin regulates MORs-cAMP/CREB-BDNF pathways in opioid-dependent models and improves cognitive impairments. J. Ayurveda Integr. Med..

[79] Williams C.M., El Mohsen M.A., Vauzour D., Rendeiro C., Butler L.T., Ellis J.A., Whiteman M., Spencer J.P.E. (2008). Blueberry-induced changes in spatial working memory correlate with changes in hippocampal CREB phosphorylation and brain-derived neurotrophic factor (BDNF) levels. Free. Radic. Biol. Med..

[80] Hardy J., Selkoe D.J. (2002). The Amyloid Hypothesis of Alzheimer’s Disease: Progress and Problems on the Road to Therapeutics. Science.

[81] LaFerla F.M., Green K.N., Oddo S. (2007). Intracellular amyloid-β in Alzheimer’s disease. Nat. Rev. Neurosci..

[82] Cozachenco D., Ribeiro F.C., Ferreira S.T. (2023). Defective proteostasis in Alzheimer’s disease. Ageing Res. Rev..

[83] Polling S., Ormsby A.R., Wood R.J., Lee K., Shoubridge C., Hughes J.N., Thomas P.Q., Griffin M.D.W., Hill A.F., Bowden Q. (2015). Polyalanine expansions drive a shift into α-helical clusters without amyloid-fibril formation. Nat. Struct. Mol. Biol..

[84] Fleming A., Bourdenx M., Fujimaki M., Karabiyik C., Krause G.J., Lopez A., Martín-Segura A., Puri C., Scrivo A., Skidmore J. (2022). The different autophagy degradation pathways and neurodegeneration. Neuron.

[85] Macedo D., Jardim C., Figueira I., Almeida A.F., McDougall G.J., Stewart D., Yuste J.E., Tomás-Barberán F.A., Tenreiro S., Outeiro T.F. (2018). (Poly)phenol-digested metabolites modulate alpha-synuclein toxicity by regulating proteostasis. Sci. Rep..

[86] Wang B., Tang X., Mao B., Zhang Q., Tian F., Zhao J., Cui S., Chen W. (2024). Anti-aging effects and mechanisms of anthocyanins and their intestinal microflora metabolites. Crit. Rev. Food Sci. Nutr..

[87] Li H., Zheng T., Lian F., Xu T., Yin W., Jiang Y. (2022). Anthocyanin-rich blueberry extracts and anthocyanin metabolite protocatechuic acid promote autophagy-lysosomal pathway and alleviate neurons damage in in vivo and in vitro models of Alzheimer’s disease. Nutrition.

[88] Francis Y.I., Fà M., Ashraf H., Zhang H., Staniszewski A., Latchman D.S., Arancio O. (2009). Dysregulation of Histone Acetylation in the APP/PS1 Mouse Model of Alzheimer’s Disease. J. Alzheimer’s Dis..

[89] Li S., Lei Z., Sun T. (2023). The role of microRNAs in neurodegenerative diseases: A review. Cell Biol. Toxicol..

[90] Liu X., Jiao B., Shen L. (2018). The Epigenetics of Alzheimer’s Disease: Factors and Therapeutic Implications. Front. Genet..

[91] Day J.J., Sweatt J.D. (2011). Epigenetic Mechanisms in Cognition. Neuron.

[92] Qazi T.J., Quan Z., Mir A., Qing H. (2018). Epigenetics in Alzheimer’s Disease: Perspective of DNA Methylation. Mol. Neurobiol..

[93] Wang Y., Zhao L., Wang D., Huo Y., Ji B. (2016). Anthocyanin-rich extracts from blackberry, wild blueberry, strawberry, and chokeberry: Antioxidant activity and inhibitory effect on oleic acid-induced hepatic steatosis in vitro. J. Sci. Food Agric..

[94] Celik E., Sanlier N. (2019). Effects of nutrient and bioactive food components on Alzheimer’s disease and epigenetic. Crit. Rev. Food Sci. Nutr..

[95] Sicilia A., Scialò E., Puglisi I., Lo Piero A.R. (2020). Anthocyanin Biosynthesis and DNA Methylation Dynamics in Sweet Orange Fruit [*Citrus sinensis* L. (Osbeck)] under Cold Stress. J. Agric. Food Chem..

